# Nanoparticle Interactions with the Immune System: Clinical Implications for Liposome-Based Cancer Chemotherapy

**DOI:** 10.3389/fimmu.2017.00416

**Published:** 2017-04-06

**Authors:** Ninh M. La-Beck, Alberto A. Gabizon

**Affiliations:** ^1^Department of Immunotherapeutics and Biotechnology, Texas Tech University Health Sciences Center School of Pharmacy, Abilene, TX, USA; ^2^Oncology Institute, Shaare Zedek Medical Center, Hebrew University-School of Medicine, Jerusalem, Israel

**Keywords:** liposome, immunosuppression, oncology, doxorubicin, alendronate, immune modulation

## Abstract

The development of stable and long-circulating liposomes provides protection of the drug cargo from degradation and increases tumor drug delivery, leading to the design of liposome formulations with great potential in cancer therapy. However, despite the sound pharmacologic basis, many liposomal as well as other nanoparticle-based drug formulations have failed to meet regulatory criteria for approval. The question that arises is whether we have missed key liposome–host interactions that can account for the gap between the major pharmacologic advantages in preclinical studies and the modest impact of the clinical effects in humans. We will discuss here the nanoparticle–immune system interactions that may undermine the antitumor effect of the nanodrug formulations and contribute to this gap. To overcome this challenge and increase clinical translation, new preclinical models need to be adopted along with comprehensive immunopharmacologic studies and strategies for patient selection in the clinical phase.

## Introduction

**Figure 1:**
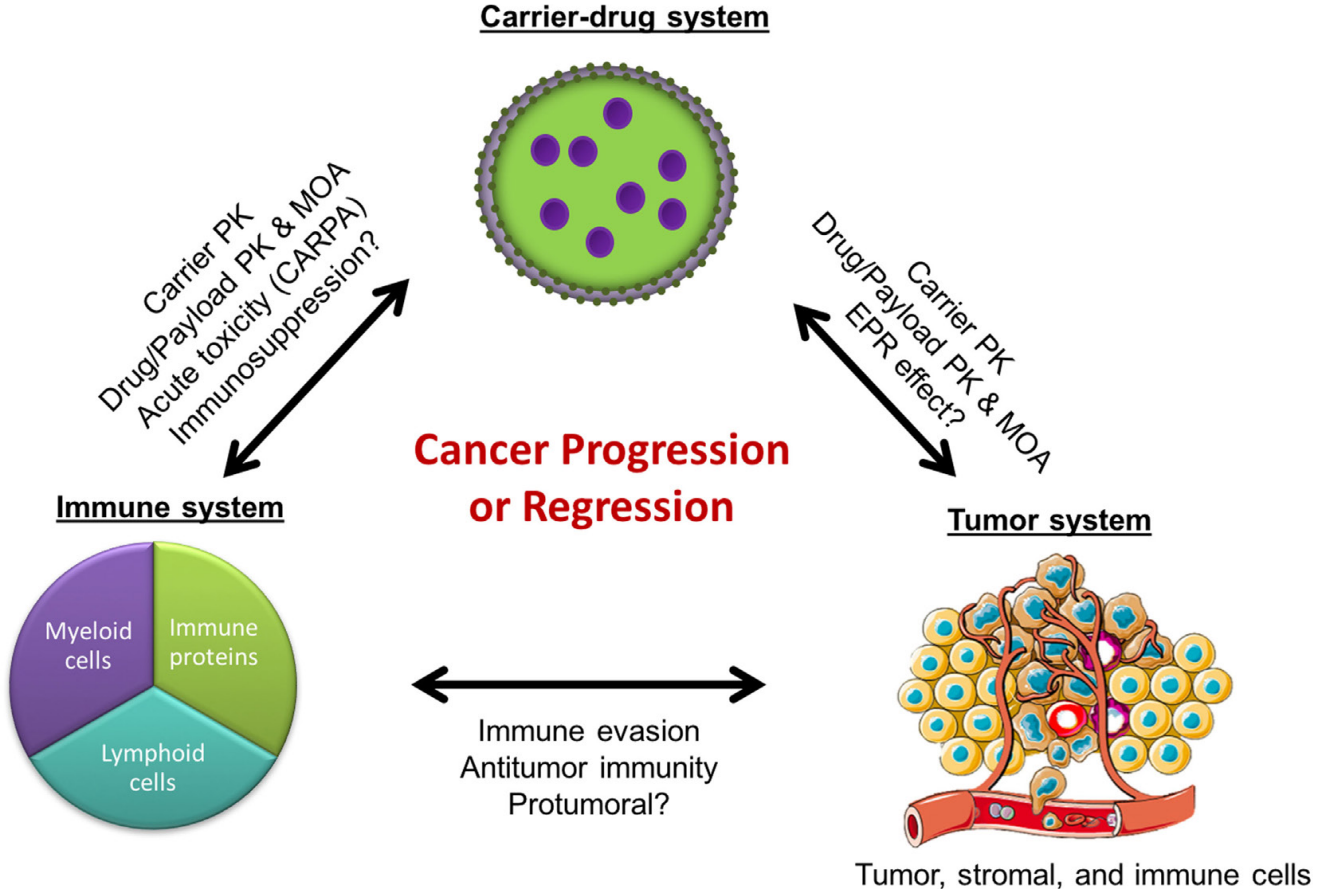
**Cancer regression or progression as a result of complex interactions between the immune, tumor, and carrier-drug systems**. PK, pharmacokinetics; MOA, mechanisms of action; EPR, enhanced permeability and retention; CARPA, complement activation-related pseudoallergy.

In the field of nanomedicine, liposomes are the most common nanocarrier platforms among the approved agents and those under clinical investigation. The development of stable and long-circulating liposomes provides protection of the drug cargo from degradation (1) and increases tumor drug delivery by exploiting the enhanced permeability and retention (EPR) effect (2). This has led to the design of liposome formulations with great potential in cancer therapy. However, despite the sound pharmacologic basis, many of the liposomal drugs as well as other nanoparticle-based drug formulations have failed to meet regulatory criteria for approval or have shown modest effects in phase 3 clinical studies (3–8). In fact, some of the approvals have been based on reduced toxicity rather than increased efficacy. A recent meta-analysis of 14 randomized clinical trials that directly compared the anticancer efficacy of liposomal cytotoxic chemotherapy to their conventional “free” drug formulation found that liposome encapsulation of drugs did not improve objective response rates, progression-free survival, or overall survival in cancer patients (6). This highlights a major roadblock: despite the pharmacological advantages of improved drug delivery, liposome-mediated therapies have largely failed to increase anticancer efficacy over conventional formulations. Yet, they remain attractive delivery platforms due to their ability to considerably improve drug tolerability and decrease toxicity in cancer patients. The question that arises is whether we have missed some liposome–host interactions that can account for the gap between the major pharmacologic advantages in preclinical studies, on the one hand, and the modest impact of the clinical effects in humans, on the other hand. We will discuss here the possibility that some nanoparticle–immune system interactions may undermine the antitumor effect of the nanodrug formulations and contribute to this gap (Figure 1).

## Clinical Evidence of Nanoparticle–Immune Interactions

Nanoparticles are known to interact with the innate immune system, including the complement system and the mononuclear phagocyte system (MPS) to varying extents, and these interactions with the immune system have significant clinical consequences. They can activate circulating complement proteins (9, 10), leading sometimes to an infusion reaction known as complement activation-related pseudoallergy (CARPA). Blood complement activation by pegylated liposomal doxorubicin (PLD) has been implicated as the cause of acute infusion reactions in cancer patients (11). Importantly, it has been reported that polymer nanoparticles that activate the complement system can promote tumor growth through generation of C5a (12), a complement anaphylatoxin that has been shown to induce tumor growth *via* recruitment of myeloid-derived suppressor cells (MDSCs) (13). Nonetheless, the clinical relevance of this is unclear since the nanoparticles in these studies were engineered to activate complement, whereas all clinically relevant nanoparticles have been designed to limit complement activation. Furthermore, while most nanoparticles still activate blood complement proteins to some extent, it is not known whether and how much they activate complement in the tumor microenvironment.

In addition to the complement system, the MPS is known to interact extensively with nanoparticles. The MPS is comprised primarily of monocytes and macrophages in the blood, spleen, and liver. These cells, especially when activated, have high phagocytic capacity and normally function to scavenge and clear cellular debris, damaged/apoptotic cells, and foreign materials including bacteria, viruses, and nanoparticles. In cancer patients, peripheral blood monocyte count was found to correlate with PLD clearance rates: a decrease in monocyte count was associated with a decrease in clearance rate (14), suggesting a direct relationship between functionality of the MPS and nanoparticle drug pharmacokinetics (PK). This was further supported by a subsequent clinical study showing that phagocytic capacity of the MPS, as measured by *in vivo* technetium sulfur colloid uptake, correlated with liposome clearance rates in patients (15).

In addition to clearance of debris and foreign particles, the MPS also regulates the adaptive immune response through the antigen-presentation functions of dendritic cells and can stimulate or inhibit T cell proliferation and cytokine responses (16). Hence, it is in theory possible for nanoparticle interactions with the innate immune system to impact adaptive immunity. For example, PLD has been reported to suppress patient sensitization and allergic reactions to carboplatin in patients receiving a combination of both the drugs (17), suggesting that PLD liposomes may have direct or indirect suppressive effects on lymphocytes. However, overall, the interactions between nanoparticles and the adaptive immune system have received comparatively little attention in clinical trials of carrier-mediated anticancer drugs. One application of nanomedicine that is focused on the adaptive immune system is the use of nanoparticles as vehicles to deliver antigens and boost their immunogenicity as vaccines. The particles used in this context are designed to exploit uptake by antigen-presenting cells, mostly dendritic cells, and thereby enabling induction of antigen-specific adaptive immunity (18). While it is difficult to generalize findings from this field to the cancer drug delivery field, it is clear that nanoparticles have the potential to modulate immune status at the interface between the innate and adaptive immune system.

When considering immunomodulatory carrier-mediated effects, one liposome component that stands out is the polyethylene glycol (PEG) polymer coating used in many liposomal formulations (19). PEG is well known for reducing immunogenicity (20) and for its immunocamouflage properties (21). PEG-modification (pegylation) is generally believed to diminish complement activation responses and evade clearance by the immune system, thereby enabling long circulation of nanoparticles (22). However, PEG has also been found to induce complement activation in this scenario as well (23). In preclinical models, accelerated blood clearance (ABC) of subsequent doses of pegylated liposomes was observed in animals, and this has been associated with the production of anti-PEG antibodies (24). However others have attributed the ABC phenomenon to non-specific binding of PEG to complement proteins, leading to complement activation and subsequent clearance of the particles (25). The clinical relevance of these findings is unclear. Preexisting anti-PEG antibodies may be present in 56–72% of the general population (26), yet the ABC phenomenon has not been reported in humans. Interestingly, the opposite effect, decreased clearance of subsequent doses of doxorubicin-loaded pegylated liposomes, was seen in cancer patients (27). Nonetheless, these reports strongly support a role for PEG in the immunopharmacology of liposome-mediated drugs and highlight critical gaps in current understanding of the mechanisms of PEG–immune interactions in the setting of cancer drug delivery.

## Potential Impact on Cancer Progression and Regression

It is evident that nanoparticle interactions with the immune system affect drug tolerability, immunogenicity, and PK. However, their impact on anticancer efficacy remains to be elucidated. The tumor microenvironment is complex, and tumors are often infiltrated by immune cells such as monocytes, macrophages, and MDSCs, which are believed to have protumoral functions through their inhibition of T cell antitumor responses and enhancement of tumor angiogenesis (28, 29). Interestingly, it was recently reported that a pegylated liposomal drug carrier, similar to that used in patients, significantly enhanced tumor growth in a mouse model of HPV-induced cancer (30). This was associated with suppression of antitumor immunity as indicated by decreased interferon-γ production by tumor-associated macrophages and cytotoxic T cells, diminished tumor infiltration of tumor antigen-specific T cells, and decreased number of dendritic cells in tumor-draining lymph nodes. It is important to point out that these preclinical studies used an immunogenic tumor model and liposomes that did not contain any drug cargo. Therefore, the clinical relevance of these findings is uncertain since human cancers are not always immunogenic, and liposomes always have a drug payload that may affect the immune interactions as well. Yet, these data suggest that the tumor-promoting potential of the carrier may mitigate the benefits of carrier-mediated drug delivery and could partially explain why there is often an insufficient improvement in the clinical efficacy of liposomal drugs over free drugs (4, 5, 7, 31). Clearly, more preclinical and clinical research efforts are needed to elucidate the precise mechanisms by which nanoparticles interact with immune cells, the consequences of this interaction on cancer progression, and the impact of the drug cargo as well as the tumor immunologic milieu on these carrier–immune interactions.

Importantly, recent studies combining liposomal doxorubicin with immune modulatory drugs in mouse models of cancer suggest that this strategy can overcome carrier-associated immunosuppression and even result in synergistic anticancer effects. Co-encapsulation of doxorubicin with alendronate, an amino-bisphosphonate with immune stimulatory effects, in a pegylated liposome showed greater anticancer efficacy than PLD in immunocompetent mouse tumor models (32). Likewise, combining PLD with immune checkpoint inhibitors targeting the PD-1 and CTLA-4 pathways also resulted in enhanced anticancer efficacy as compared to PLD in immunocompetent mice (33). Notably, these combination treatment approaches failed to show improved efficacy over PLD in immunocompromised mice, supporting the pivotal role of the immune system in determining the efficacy of the nanomedicine-based anticancer treatments (33). This host effect was much less important in the case of some low molecular weight drugs such as doxorubicin and gemcitabine. Moreover, all but one mouse with complete tumor response following PLD treatment rejected a rechallenge with the same tumor cells, indicating that they had become immunized (33). Since these observations were done with a cargo of doxorubicin, a drug that is well known for often leading to immunogenic tumor cell death (34), we cannot make extrapolations to other nanomedicines. Nonetheless, clinical trials examining the anticancer efficacy of combined liposomal chemotherapy with immune checkpoint inhibitor antibodies are clearly warranted.

## Are We Using the Right Preclinical Models?

If indeed the liposome carriers and/or their drug payload have immunomodulatory effects, then the use of immunocompetent mice is critical for observing the full pharmacologic effect. This entails the use of mouse syngeneic tumor models, since allogeneic xenografts such as human tumors would not grow in immunocompetent mice. In the last two decades, there has been a shift to models based on human tumor xenografts implanted in immunocompromised mice whether athymic mice (lacking T cells), SCID mice (lacking T and B cells), or Beige mice (lacking natural killer lymphocytes), the reasoning being that human tumors will be more predictive of the activity of new drugs in the clinic. This is probably the case for low molecular weight drugs, but, when more complex systems are used such as nanomedicines, the risk of overlooking an important interaction with the immune system may override any advantage that a human tumor model may offer over a syngeneic tumor, as mentioned above (32, 33).

Among the various immunocompetent mouse models, there are important distinctions in global immune status (e.g., balance of Th1–Th2 cytokines or M1–M2 macrophages) that may affect nanoparticle disposition. The Th1-dominant mouse strains such as C57BL/6 and B10D2 have been reported to have slower rates of clearance of pegylated 300-nm cylindrical hydrogel nanoparticles than the Th2-dominant strains such as BALB/c and DBA/2 (35). These differences in clearance were correlated with increased M1 macrophage polarization and lower particle uptake in Th1-dominant strains, and increased M2 macrophage polarization and higher particle uptake in the Th2-dominant strains. Likewise, when silica nanoparticles were tested *in vitro* with THP1 cells, an immortalized human monocytic cell line, alternatively activated (M2-like) THP1 cells demonstrated higher nanoparticle uptake than classically activated (M1-like) THP1 cells (36). In contrast, another study found that the uptake of pegylated or non-pegylated spherical polystyrene nanoparticles by murine bone marrow-derived macrophages is highest in classically activated M1 macrophages, followed by alternatively activated M2 macrophages, and lowest in unactivated M0 cells (37). There is likely no single ideal mouse model, and selection should take into consideration the clinical immune characteristics of the host, type of cancer, and type of nanoparticle that are being modeled.

To counter the shortfalls of immunocompromised mice as hosts of human tumor xenografts, humanized mouse models, in which the immune system of SCID mice is reconstituted with human bone marrow, have been developed and are being increasingly used particularly in cancer studies that involve immunotherapeutic approaches (38). One step further is the use of patient-derived tumor xenografts (39), instead of the commonly used human tumor cell lines. The testing of nanomedicines in humanized mice is still lagging behind but, conceivably, may provide an important insight on the interplay of nanomedicines with the human immune system.

Another major tumor model factor affecting the testing of nanomedicines is the choice between primary tumor implants and metastatic tumors. Given that the EPR effect is the main mechanism for selective accumulation of nanomedicines in tumors (2), tumor sites with high or poor EPR will respond differently to nanomedicines. This is because the degree of EPR appears to be dependent on the tumor type and on the site of tumor growth (40). Primary tumor implants, particularly those inoculated subcutaneously, recruit new blood vessels for growth and usually demonstrate high EPR. Less well known is the degree of EPR of orthotopically implanted tumors. However, when hematogenous metastases occur either by detachment from primary tumors or by intravenous injection, tumor cells form multiple and separate colonies in lungs and other organs. These tumor colonies grow around well-developed and mature blood vessels of the host organ and often derive their blood supply by a process known as co-option of normal blood vessels, which results in blood vessels less permeable and less responsive to antiangiogenic treatments and, consequently, less likely to display the EPR effect (41, 42). Clearly, given that the clinical challenge is to treat patients with metastases, an effort should be made to include metastatic tumor models in the testing of nanomedicines to achieve a better prediction of their potential performance in human cancer (43).

## Are We Using the Right Clinical Trial Designs and Assessments?

Most, if not all, clinical studies with liposome-based chemotherapy and other nanomedicines have been performed without any attempt to select for those cancer patients with tumors that display high liposome uptake. This may have contributed to some disappointing clinical results. For example, in the phase 3 study of PLD against doxorubicin single agent in metastatic breast cancer, no survival advantage was observed (4) despite the clear superiority of PLD in animal tumor models. Recent studies in mouse models using radionuclide SPECT imaging with In^111^-labeled liposomes and PET imaging with Zr^89^-labeled liposomes admixed with PLD have observed that higher tumor uptake of liposomes correlates with greater antitumor activity at the individual level (44, 45). These studies also show a correlation between tumor uptake and tumor microvessel density and reveal remarkable heterogeneity in liposomal tumor accumulation. Based on the preclinical data, one would predict that the efficacy results of clinical studies with stable nanomedicines such as PLD could have been much improved by selection of a patient population with high EPR tumors. By imaging the fate of the nanoparticles, the EPR-dependent tumor uptake of the drug payload can be predicted in each specific case and correlated with the clinical response. This would provide direct clinical data to determine whether or not selecting patients based on the EPR characteristics of their tumor could lead to improved therapeutic benefit of PLD or any other nanoparticle-based therapy (46, 47).

Another aspect of nanomedicines that has not been addressed in clinical studies is the interaction with the immune system. Future clinical studies should incorporate immunopharmacologic tests to gain an insight on these interactions. With liposomes, immunomodulation can occur at least at two different levels:

1. The CARPA reaction has been described in the preceding section of this article. While nanoparticle-induced blood complement activation may be common in patients, CARPA seldom manifests clinically in patients treated with PLD when infusion protocols are carefully followed (11). Nonetheless, we do not know the incidence of subclinical complement activation and whether it may affect the immune system in the local tumor environment.
2. Macrophage function is a major factor in liposome clearance. Liver and spleen macrophages determine systemic clearance, and local tissue macrophages in tumors and other tissues are also important scavengers of extravasated liposomes. Based on this, peripheral blood monocytes have been proposed as a surrogate marker that can predict macrophage-mediated liposome clearance (14). Importantly, nanomedicines that suppress or activate macrophages either due to carrier-related effects or drug-specific effects may have direct and/or indirect consequences blunting or boosting the ultimate antitumor effect observed.

## Future Outlook

A key roadblock in the development of efficacious cancer therapies is the systemic toxicity of the majority of these agents. Drug delivery using nanoparticle carriers has been an important and valuable strategy in overcoming this challenge by dramatically improving the tolerability of anticancer drugs in patients (48). However, this approach has not yet achieved a sound improvement in clinical efficacy as predicted from its pharmacologic advantages. We propose that the immune system is a key player in the pharmacology of nanoparticle-based therapy, probably more so than for conventional low molecular weight drugs, and that new understanding of the mechanisms of immune modulation by nanoparticles and their associated drug cargo can lay the foundation for future work that will realize the full clinical potential of cancer nanomedicines.

## Conflict of Interest Statement

The authors declare that the research was conducted in the absence of any commercial or financial relationships that could be construed as a potential conflict of interest. The handling editor declared a past coauthorship with the authors and states that the process nevertheless met the standards of a fair and objective review.
